# Ice water immersion does not activate diffuse noxious inhibitory controls of spinal reflexes in sedated or anaesthetised dogs (*Canis familiaris*): a pilot study

**DOI:** 10.3389/fpain.2025.1505064

**Published:** 2025-03-10

**Authors:** J. R. Hunt, D. Knazovicky, J. Harris, S. Kelly, T. G. Knowles, J. C. Murrell, B. D. X. Lascelles

**Affiliations:** ^1^ School of Veterinary Sciences, University of Bristol, Bristol, United Kingdom; ^2^ Comparative Pain Research Laboratory, College of Veterinary Medicine, North Carolina State University, Raleigh, NC, United States; ^3^ Department of Clinical Sciences, College of Veterinary Medicine, North Carolina State University, Raleigh, NC, United States; ^4^ Division of Animal Sciences, School of Biosciences, University of Nottingham, Sutton Bonington Campus, Loughborough, United Kingdom; ^5^ Bristol Vet Specialists, Bristol, United Kingdom; ^6^ Translational Research in Pain Program, Comparative Pain Research and Education Centre, Department of Clinical Sciences, College of Veterinary Medicine, North Carolina State University, Raleigh, NC, United States; ^7^ Center for Translational Pain Research, Department of Anesthesiology, Duke University, Durham, NC, United States; ^8^ Thurston Arthritis Center, UNC, Chapel Hill, NC, United States

**Keywords:** DNIC, canine, ice-water bath, CPM, pain

## Abstract

**Introduction:**

Diffuse noxious inhibitory controls (DNIC) may be impaired in human subjects with osteoarthritis (OA) pain. Spontaneously occurring OA in dogs is considered a valuable model of human OA; however, methodology for assessing DNIC in dogs has not been fully developed. The aim of this study was to develop a suitable DNIC protocol using ice water immersion, similar to protocols used in humans.

**Objective:**

This study objective was to create an experimental protocol for inducing DNIC in sedated or anesthetized dogs, ensuring it has face validity for future assessments of DNIC in studies involving the spontaneous canine OA model. We hypothesized that inducing DNIC in healthy dogs would result in a reduced electromyographic (EMG) response to a specific nociceptive stimulus.

**Methods:**

Electromyographic (EMG) responses of the cranial tibial muscle to test electrical stimuli and interdigital skin temperature were recorded in seven healthy dogs before and during a 20-min duration conditioning ice water immersion of the distal forelimb. The protocol was repeated for each dog using three different states: sedation with acepromazine or alfaxalone or anaesthesia with alfaxalone.

**Results:**

Ice water immersion caused a decrease of interdigital skin temperature in dogs in all three groups with the nadir (4.9–13.6°C) at 10 min following immersion. Skin temperatures remained significantly higher (*p* = 0.018) in alfaxalone sedated compared to acepromazine sedated dogs and returned to baseline more quickly than in acepromazine sedated dogs. Magnitudes of EMG responses were significantly larger in acepromazine sedated dogs compared to alfaxalone treated dogs (*p* < 0.001). DNIC was not induced, as the EMG magnitude did not significantly change over time for either the early (*p* = 0.07) or late responses (*p* = 0.27), and no significant interactions were observed between time and anaesthetic state in relation to EMG magnitude.

**Conclusion:**

Our data suggest that a cold conditioning stimulus failed to elicit DNIC. It is possible that the magnitude of the conditioning stimulus was not sufficient to recruit DNIC in dogs.

## 1 Introduction

Diffuse noxious inhibitory controls (DNIC) represent an endogenous descending inhibition of nociceptive central nervous system activity induced by a heterotopic noxious (“conditioning”) stimulus (1, 2). DNIC is assessed experimentally in man by measuring the decrease in pain sensation, increase in pain threshold (3), or reduction in nociceptive withdrawal reflex (NWR) magnitude (4) to a noxious (“test”) stimulus, following application of the conditioning stimulus. Impaired DNIC is an epiphenomenon associated with an altered balance of descending modulation and is maintained in a number of conditions, including osteoarthritis (OA) (5, 6). Impaired DNIC may reflect an altered balance between pro- and anti-nociceptive descending pathways towards less inhibition and/or greater facilitation of pain transmission at the spinal level (7). Spontaneously occurring canine OA has been proposed as a model for further investigation of, and trial of therapeutics for, the human condition (8–10). Analogous to psychophysical testing in man, there is evidence of widespread central sensitisation to nociceptive stimuli in dogs affected by spontaneous hindlimb OA (11). The clinical phenomenon of secondary hyperalgesia may result from segmental mechanisms of central sensitisation within the dorsal horn of the spinal cord, or may alternatively result from impaired descending inhibition or descending facilitation of nociception (12, 13), or a combination of both. DNIC has been recently explored in both healthy pain free dogs and dogs with OA pain (14, 15) using mechanical stimuli as the conditioning stimulus (blunt probes or blood pressure cuffs). In humans, numerous stimuli have been applied in conscious volunteers, including immersion of the hand in hot water, intramuscular hypertonic saline, ischaemia, and cutaneous application of capsaicin; however the most commonly reported method of activating DNIC is immersion of the hand in iced water (the “cold pressor test”, CPT) (16, 17). Cold water pressor paradigms have not been explored in dogs. Developing a paradigm to measure DNIC in dogs is relevant because then it could be applied to the spontaneous OA-pain model in dogs (which is considered a clinically relevant model) for testing therapeutic interventions directed at normalizing the function of descending analgesic systems.

The subjective nature of the pain experience suggests that an objective measure of nociception would be of value for determining the level of inhibition resulting from a heterotopic noxious conditioning stimulus in animals. Cutaneomuscular reflexes are simple, centrally-organised responses to noxious stimuli which therefore allow changes in spinal cord excitability to be measured. For instance in man, the magnitude of the RIII reflex response (18) is decreased in association with effective conditioned pain modulation. The nociceptive withdrawal reflex (NWR) in the cranial tibial (CT) muscle, a primary mover in producing tarsal flexion in response to noxious stimulation of the toes, has previously been evaluated in dogs using electromyographic (EMG) techniques, and the magnitude shown to correlate with the stimulus strength (19). Effective DNIC-evoked inhibition of hindlimb muscle reflex responses, including the CT muscle, has been shown in other species (2, 20–22).

The aim of our exploratory, pilot work was to develop an experimental protocol for the induction of DNIC in sedated or anaesthetised dogs, which would possess face validity when assessing DNIC in future studies in the spontaneous canine OA model. We hypothesised that induction of DNIC using a cold pressor stimulus in normal dogs would cause a decreased magnitude of electromyographic (EMG) response produced by a defined noxious stimulus.

## 2 Methods

Ethical approval was obtained prior to beginning the study from the North Carolina State University Institutional Animal Care and Use Committee (IACUC 13-010-B).

### 2.1 Animals

Seven purpose bred male hound dogs (weight range 25.5–29.2 kg) were used for these experiments, following completion of NWR recordings previously described (19). Given that at the time of the study, no data existed regarding the induction of DNIC in dogs, we used a sample of 7 available dogs. Orthopedic, neurological and physical examinations by a veterinarian (BDXL), combined with hindlimb radiography, confirmed the dogs were orthopedically normal and had no detectable diseases and no painful condition(s). The dogs' accommodation comprised individual enclosures measuring 1.5 × 4.5 m, and 2.7 m high. Environmental enrichment was provided with toys that were regularly substituted. Synthetic bedding was provided. The dogs’ exercise and socialisation program consisted of two 30-min lead walks each day, outside, and two 20-min off lead play and socialisation periods each day. The dogs were maintained on a standard complete dry dog food; food was withheld for eight hours prior to administration of sedative drugs. The protocol was performed with the dogs positioned in right lateral recumbency on a synthetic padded bed, beneath which was a circulating warm water blanket and padded table top. Following completion of the experiment, carprofen (4 mg kg^−1^) (Rimadyl, Zoetis, New Jersey, USA) was administered subcutaneously, to treat any potential ongoing pain arising from the experimental procedures. Following the studies described here, the dogs were kept at North Carolina State University Animal Care Facility to participate in further research projects.

### 2.2 Placement of EMG recording electrodes

**Figure 1:**
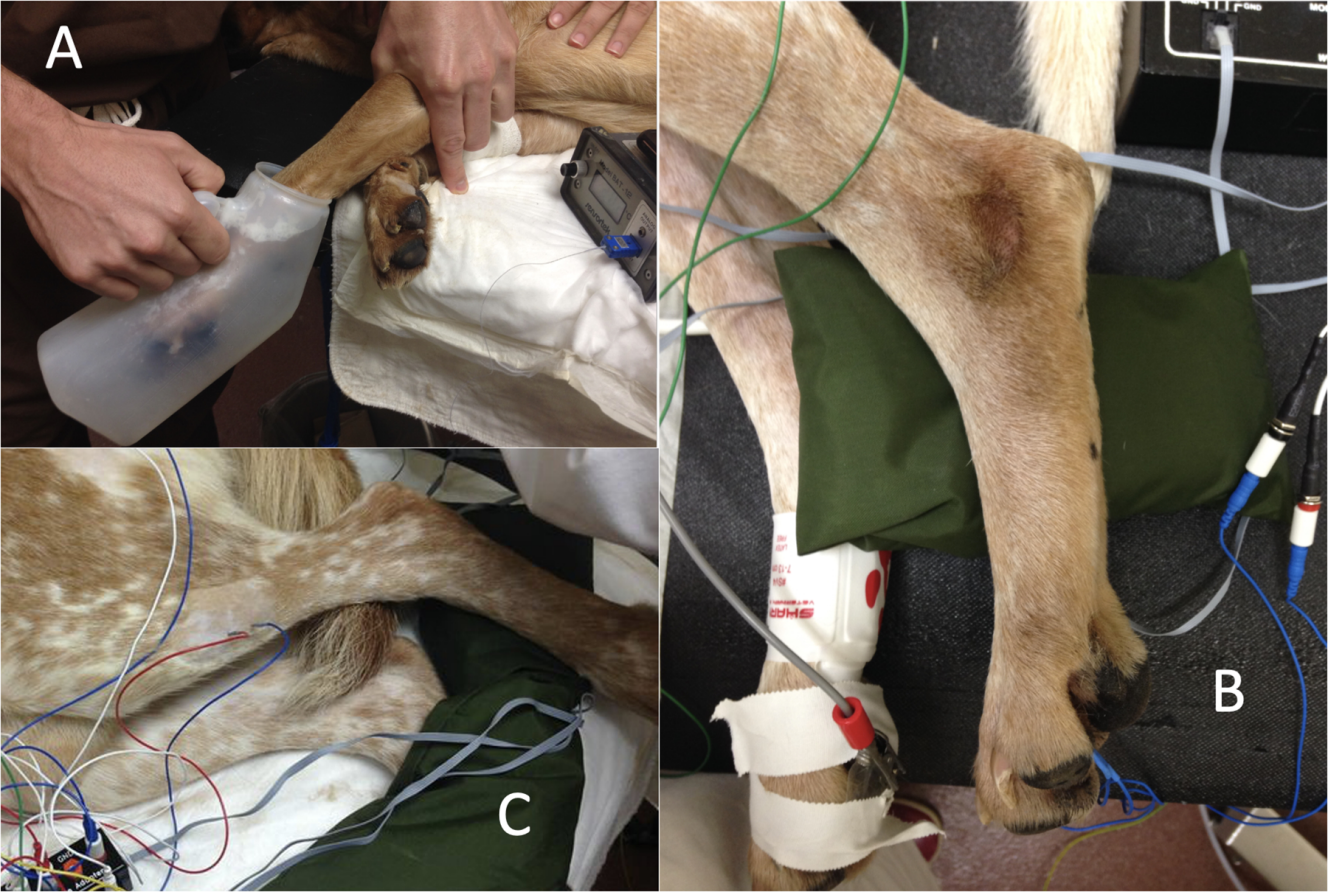
**(A)** immersion of the forelimb in ice water; **(B)** paired stimulating needle electrodes (blue leads) were introduced into the plantar dermal tissues of the distal phalanx of the fourth digit, immediately proximal to the proximal edge of the digital pad, of the left pelvic limb; **(C)** paired stainless steel needle recording electrodes (blue and red leads) were placed transcutaneously (18 mm inter-electrode distance) into the belly of the cranial tibial muscle of the left pelvic limb.

The left hindlimb was supported perpendicularly to the tabletop by resting the pes on a sandbag. Paired stainless steel needle recording electrodes (disposable subdermal needle electrode 12 × 0.40 mm, Natus Neurology Inc. Middleton, WI, USA) were placed transcutaneously (18 mm inter-electrode distance) into the belly of the cranial tibial muscle of the left pelvic limb. A ground electrode was placed subcutaneously, dorsal to the dorsal spinous processes of L4 to L6. Recording electrodes were connected to an analogue to digital converter (Powerlab 4/35, AD instruments, Oxford, UK) via separate differential amplifiers (DAM50, World Precision Instruments, Herts, UK) which applied a low pass filter of 10 Hz, high pass filter of 1 KHz, and gain of 1,000 to the signal. The resulting digital signal was captured and recorded using Labchart 8 software (AD instruments, Oxford, UK) run on a Toshiba Tecra R850 (Toshiba Europe, 41460 Neuss, Germany) laptop, utilising Windows 7 operating system (Microsoft Corp., USA). Further illustration of the recording electrode arrangement is available (19) and Figure 1 shows the experimental set up visually.

### 2.3 Sedation and anaesthesia

Dogs underwent the DNIC-testing procedure described below in each of three states: acepromazine sedation, alfaxalone sedation or alfaxalone anaesthesia. A wash-out period of 3 days between the different methods of sedation was used. In all states, dogs were administered acepromazine (Acepromazine Maleate Injection, Boehringer Ingelheim Vetmedica, Missouri, USA) 0.03 mg kg^−1^ intramuscularly 30 min prior to being instrumented for EMG recording. For the acepromazine sedation group no additional drugs were used. The alfaxalone sedation state was produced by administration of an intravenous bolus of 1 mg kg^−1^ alfaxalone (Alfaxan®, Jurox, Missouri, USA), 30 min following acepromazine. Sedation (sufficient to cause the dogs to remain in lateral recumbency, remain unresponsive to quiet auditory stimuli, and exhibit decreased muscle tone but maintain laryngeal reflexes) was maintained by continuous intravenous infusion of alfaxalone (range 0.044–0.08 mg kg^−1^ min^−1^). Dogs in this state were provided with supplemental oxygen (1–2 L min^−1^) via a close-fitting facemask. The alfaxalone anaesthesia state was produced by IV administration of 1–2 mg kg^−1^ alfaxalone, 30 min following acepromazine, injected over a period of 60 s until adequate conditions for orotracheal intubation were achieved. Anaesthesia (sufficient to maintain orotracheal intubation with a cuffed endotracheal tube through which oxygen was delivered via a circle rebreathing system) was maintained by a continuous intravenous infusion of alfaxalone. The rate of alfaxalone infusion (range 0.075–0.1 mg kg^−1^ min^−1^) was adjusted to preserve a slow palpebral reflex. Following completion of the DNIC protocol, alfaxalone infusions were discontinued and dogs were monitored during recovery to the point that they were able to walk unaided, at which point they were transported back to their kennel.

### 2.4 DNIC protocol

Paired stimulating needle electrodes (disposable subdermal needle electrode 12 × 0.40 mm, Natus Neurology Inc. Middleton, WI, USA) were introduced into the plantar dermal tissues of the distal phalanx of the fourth digit, immediately proximal to the proximal edge of the digital pad, of the left pelvic limb (see Figure 1). Electrical test stimuli (5 × 5 mA, 1 ms pulse, 100 Hz) were delivered using a constant current stimulator from an isolated 100 V source (Stimulus isolator FE180, AD instruments, Oxford, UK) via the stimulating electrodes and EMG responses recorded from the ipsilateral cranial tibial muscle at time points (T) -15, -10, -5, 0, 5, 10, 15, 20, 25, and 30 min. The means of early (0–100 ms) and late (100–500 ms) EMG responses recorded at time points -15, -10, -5, and 0 were calculated and denoted as control. Between T0 and T20, a conditioning stimulus comprising immersion of the manus and carpus (to the level of the radiocarpal joint) in iced water (0–4°C) was applied to the distal left forelimb, which had had the hair clipped from it and the skin degreased with soap. Interdigital skin temperature measurements were recorded every 2 min using a thermocouple by briefly removing the foot from the ice water bath to measure the temperature. The dogs were monitored for adverse events related to cold exposure such as pain, or skin or claw damage for 7 days following the experiments.

### 2.5 EMG analysis and statistical approach

Post recording, 10 Hz digital filtering was applied to the EMG traces, to further decrease movement artefact. The early and late phases of the rectified EMG response were measured as previously described (19).

The integral of the rectified EMG response was extracted for each stimulus. Natural log (ln)[integral] values were subsequently analysed using the statistics package MLwiN (23) which allowed the repeated measures structure of the data to be properly accommodated within the analyses. The statistical model was based on a structure consisting of an individual measurement within each anaesthetic state, within dog, and then the effect of anaesthetic state and conditioning stimulus was tested within a general linear model. To test for a non-linear relationship a series of polynomial terms for the stimulation response over time were specified. Terms retained within the models are reported if they were significant at *p* ≤ 0.05, with significance calculated as the ratio of the parameter estimate to its standard error and tested against a Z distribution. Overall changes to the model were tested (at *p* ≤ 0.05) against a change in the log likelihood using a Chi square distribution with degrees of freedom equal to the number of terms entered or deleted from the model. The resulting final models are presented as graphs of the ln response plotted against time, to depict the mean response to the stimulation.

## 3 Results

All of the dogs underwent the DNIC protocol in the acepromazine sedated state, four of the dogs underwent the protocol in the alfaxalone sedated state, and five in the alfaxalone anaesthetised state. Not all dogs underwent all test states of sedation due to logistical scheduling factors unrelated to this study. Arousal from sedation, necessitating increased alfaxalone infusion rate, was required in two alfaxalone sedated dogs and occurred soon after ice water immersion of the manus and carpus. Attempts to withdraw the limb from the ice water conditioning stimulus were noted in four of the acepromazine sedated dogs.

### 3.1 Effect of ice water immersion on skin temperature

**Figure 2:**
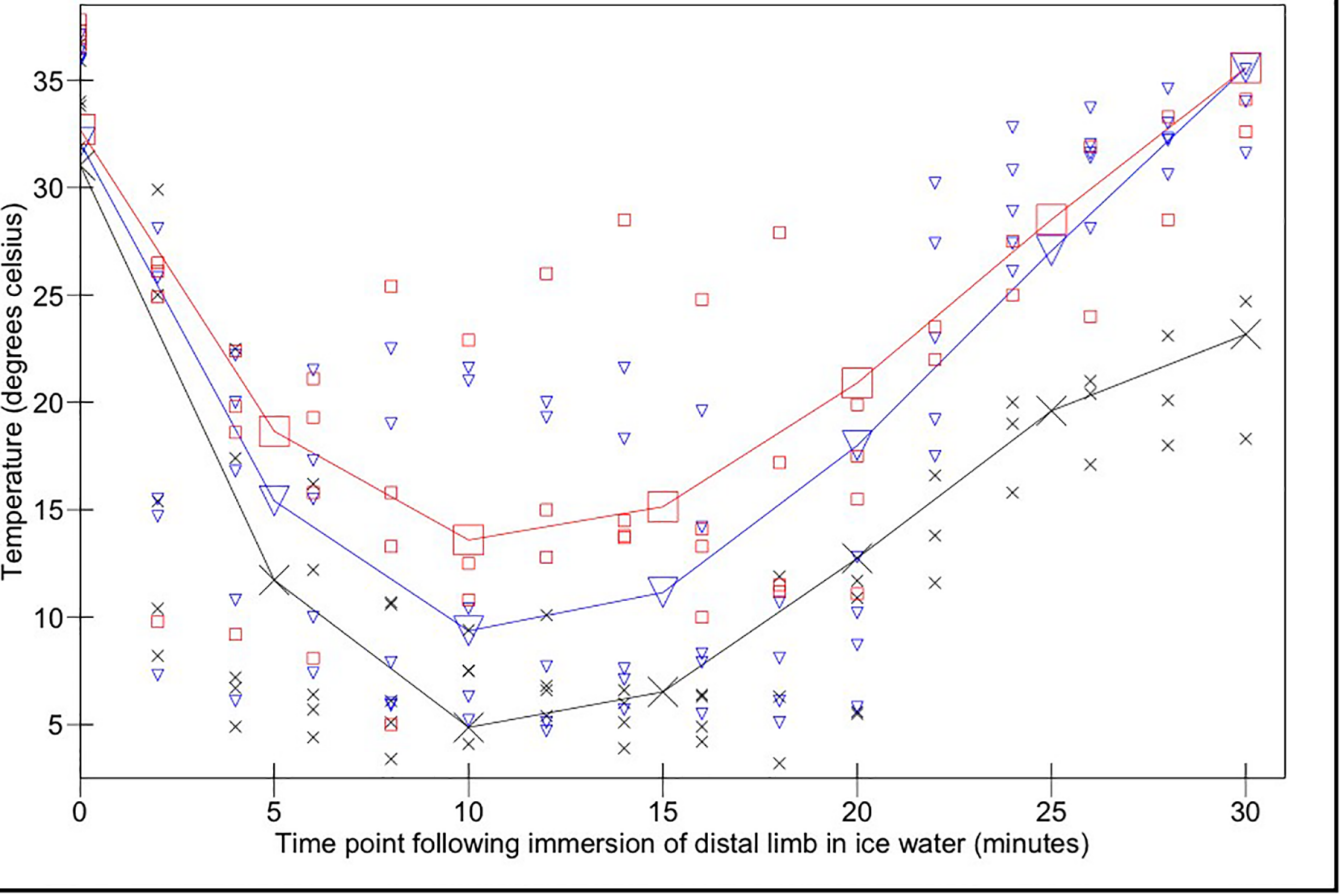
Interdigital skin temperature changes over time following immersion of the distal forelimb in ice water (from 0 to 20 min) and subsequent removal from the ice water bath (20–30 min). Acepromazine sedation is represented by X, alfaxalone anaesthesia by ▾ and alfaxalone sedation by □. Recorded values are represented by small symbols, mean results are represented by larger symbols connected by lines.

The mean interdigital skin web temperature across the three states immediately prior to immersion in ice water was 31.9°C (95% CI 27.5–36.6°C). Skin temperatures reached a minimum of 4.9°C (95% CI 1.3–8.4°C), 13.6°C (95% CI 9.8–17.3°C), and 9.4°C (95% CI 5.9–12.9°C) in acepromazine, alfaxalone sedated and alfaxalone anaesthetised states respectively following 10 min ice water immersion, after which time they began to rise despite being maintained in the water bath for a further 10 min (Figure 2).

Skin temperature measurements remained significantly higher in the alfaxalone sedated state compared to the acepromazine state during immersion (*p* = 0.018); there was no significant difference between alfaxalone groups (*p* = 0.25). Temperature changed significantly as a function of time (*p* < 0.0001). There was a significant interaction between change over time (cubed) and anaesthetic state (*p* = 0.023): ten minutes following cessation of the conditioning stimulus, skin temperatures in both alfaxalone groups had been restored to pre-conditioning temperatures [35.5°C (95% CI 30.2–40.9°C)]; skin temperatures in acepromazine sedated dogs remained significantly lower [23.2°C (95% CI 17.4–28.7°C)].

### 3.2 EMG responses from cranial tibial muscle

**Figure 3:**
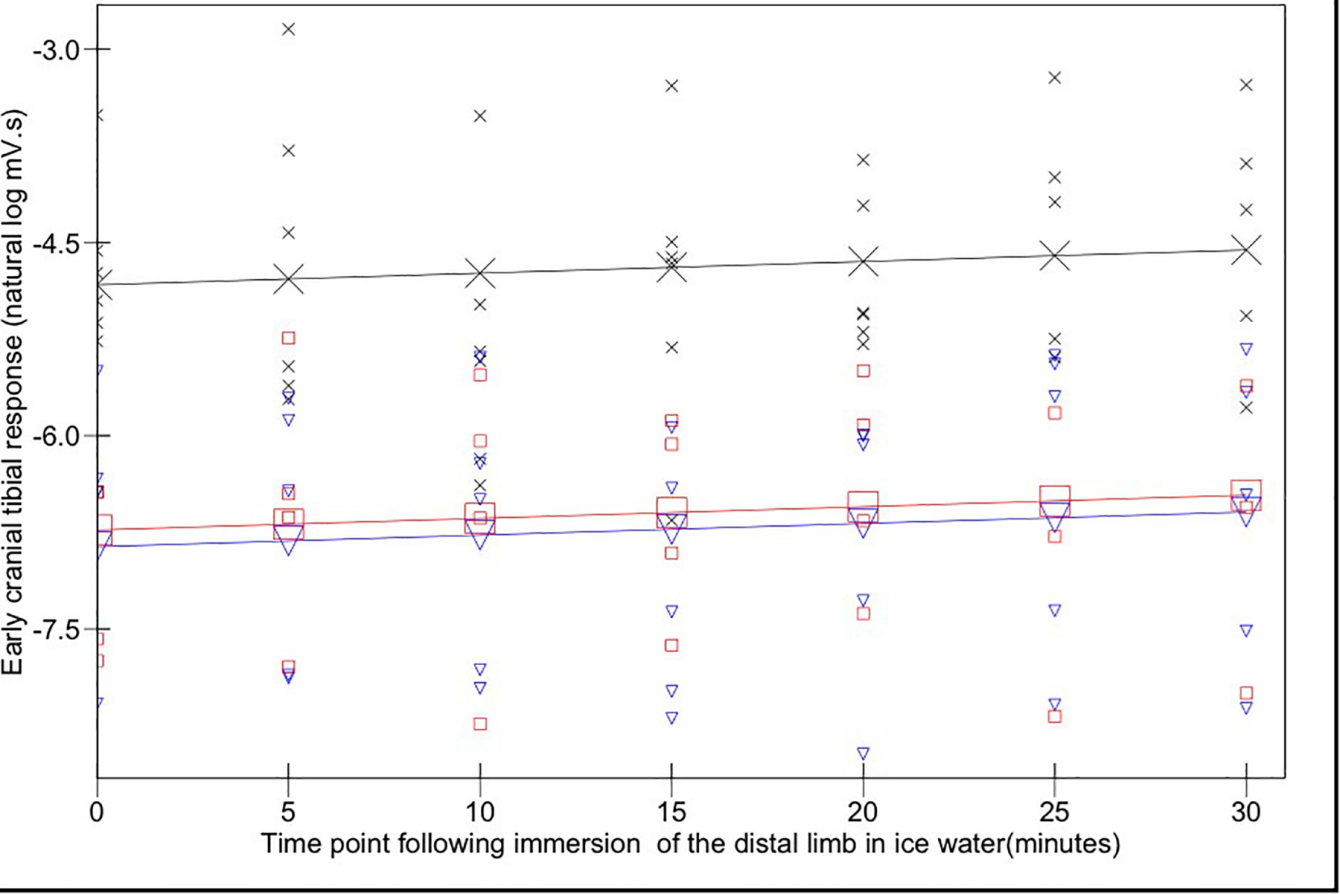
Early (0–100 ms) cranial tibial responses. Time point 0 represents the baseline (mean of responses at T-15, -10, -5, and 0). Acepromazine sedation is represented by X, alfaxalone anaesthesia by ▾ and alfaxalone sedation by □. Recorded values are represented by small symbols, mean results are represented by larger symbols connected by lines. Variation with time was not significant.

**Figure 4:**
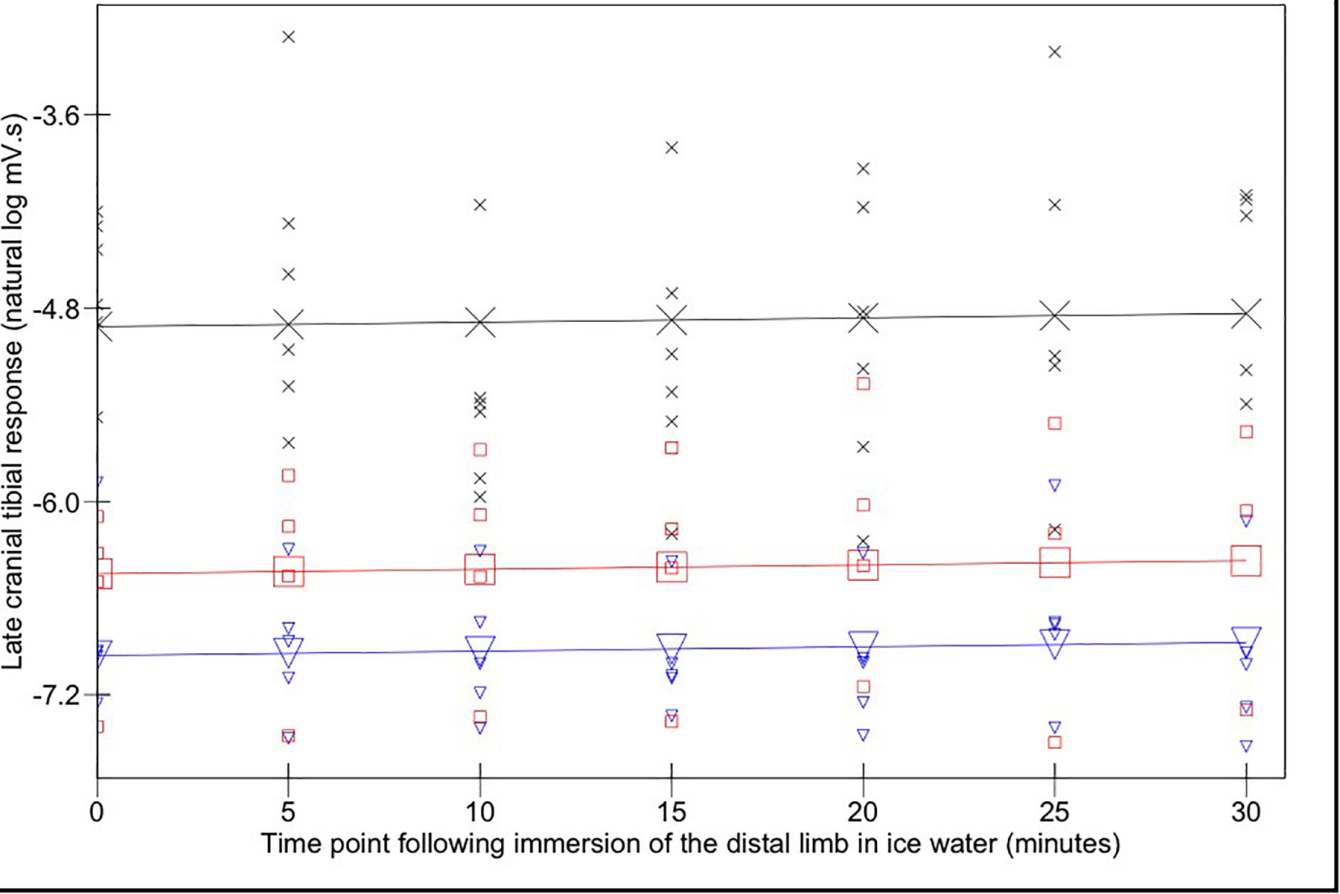
Late (100–500 ms) cranial tibial responses. Time point 0 represents the baseline (mean of responses at T-15, -10, -5, and 0). Acepromazine sedation is represented by X, alfaxalone anaesthesia by ▾ and alfaxalone sedation by □. Recorded values are represented by small symbols, mean results are represented by larger symbols connected by lines. Variation with time was not significant.

**Figure 5:**
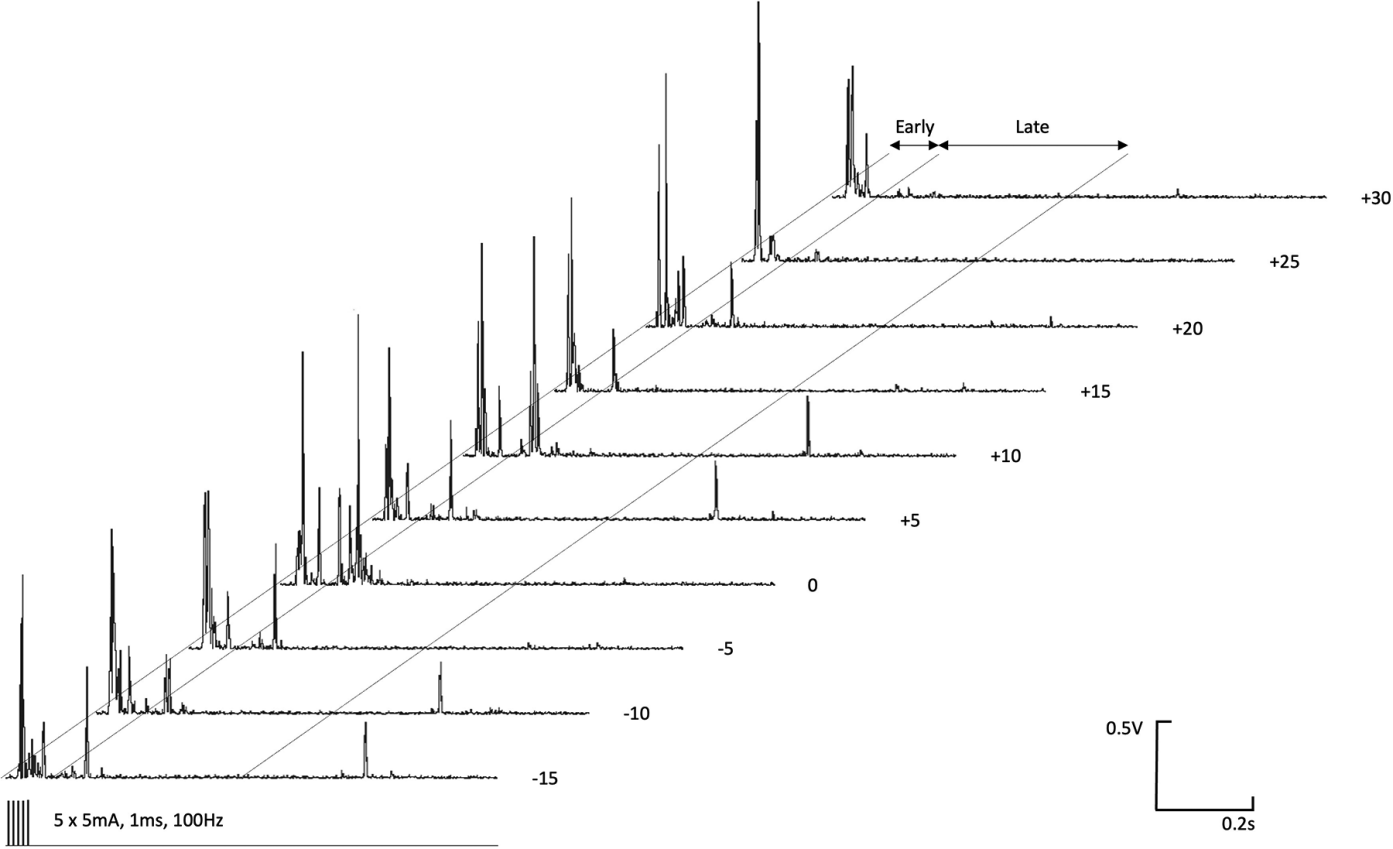
Early (0–100 ms) and late (100–500 ms) reflex responses in the ipsilateral cranial tibial muscle to high frequency electrical stimulation (5 × 5 mA, 1 ms duration, 100 Hz) of the distal phalanx of the fourth digit. Time relative to immersion of the distal left forelimb in iced water (0°C–4°C) is indicated to the right of each EMG recording.

The magnitudes of early (Figure 3) and late (Figure 4) EMG responses recorded in the acepromazine sedated dogs were significantly greater than those recorded from the dogs in both alfaxalone states (*p* < 0.001), and the magnitude of late (Figure 4) EMG responses was significantly greater in alfaxalone sedated dogs compared to alfaxalone anaesthetised dogs (*p* = 0.048). The magnitude of EMG did not vary significantly over time for the early (*p* = 0.07) or late responses (*p* = 0.27), and there were no significant interactions between time and anaesthetic state with respect to EMG magnitude. Based on EMG responses, DNIC was not induced due to the cold pressor test. Early and late reflex responses in the cranial tibial muscle to electrical stimulation of the fourth digit, with timing relative to forelimb immersion in iced water are shown in Figure 5.

No adverse effects of the protocol were seen.

## 4 Discussion

It was anticipated that exposing the distal forelimb of dogs to a noxious cold conditioning stimulus, in the form of an ice water bath, would activate a spino-bulbo-spinal pathway (DNIC) resulting in the suppression of electromyographic activity in the left pelvic limb in response to a nociceptive test stimulus. In this study, we found no suppression of electromyographic activity, suggesting DNIC was not activated. However, the presence of weak evidence for an effect on early EMG response might suggest that the study might be underpowered. Two dogs sedated with alfaxalone required an increased infusion rate due to arousal from sedation, which could have influenced the results.

Effective skin cooling by ice water immersion was recorded in all three states, and was most pronounced in acepromazine sedated dogs, however this conditioning stimulus did not appear to evoke DNIC in these normal dogs. Potential explanations for this finding are that the noxious cold stimulus was of insufficient intensity to recruit DNIC in dogs; that the magnitude of the test stimulus was too great compared to the conditioning stimulus, causing the nociceptive withdrawal response to the test stimulus to overcome DNIC effects, or that the time period during which DNIC was effective did not align with the timings of our test stimuli.

DNIC is elicited only by higher intensity (moderate and severe) noxious conditioning stimuli (24–26). Conscious, acepromazine sedated dogs responded to the conditioning stimulus by attempting to withdraw their limb from the stimulus, implying that the stimulus was perceived as aversive, however the intensity of the conditioning stimulus was challenging to assess. Increasing the intensity (24) or spatial distribution (27) of the noxious conditioning stimulus (28) can increase the recruitment of DNIC, however there is a maximum effect, at which no further increase in descending inhibition is possible (24). Application of the cold stimulus in this study to the level of the manus reflects the paradigm utilised in man (29) but as has been shown recently for heat stimuli, increasing the surface area exposed to the stimulus may result in spatial summation (27), and a perhaps more effective conditioning stimulus. Different conditioning stimuli have been reported to produce varying degrees of DNIC, but cold stimuli appear to produce the most robust DNIC responses in humans (30, 31).

Investigations of conditioned pain modulation in man have employed a test stimulus which produces a subjective pain experience of magnitude 60 out of 100 (24), whilst effective conditioning stimuli have produced subjective pain experiences of magnitude 31.57 ± (SD) 9.56 (moderate intensity) and 58.1 ± (SD) 11.43 (severe intensity) (24). It is possible that the test stimulus used in this study (electrical stimuli) was of a magnitude greater than the equivalent of 60 out of 100 in humans, and generated a more robust nociceptive withdrawal response than has been investigated in previous studies, suppression of which may have therefore been more difficult to induce. However the stimulus chosen (5 × 5 mA, 100 Hz) produced a response approximating 50%–60% of maximum in our previous findings (19), therefore would have been expected to be of an appropriate magnitude to be applied as a test stimulus.

The cold pressor test in man is reported to provoke 90%–95% of the total reduction in skin temperature within the first minute (29). Mourot and others (32) reported skin temperatures of 19 male subjects undergoing CPT, which were measured as 14.3 ± (SD) 4.6°C and 13.4 ± (SD) 5.1°C following 2 and 3 min immersion respectively. We observed a much slower decline in skin temperatures, compared to studies of CPT in man, however, at their nadir, the skin temperatures recorded in the present study reached similar temperatures to those reported by Mourot and others (32). Therefore, if skin temperature alone is the governing factor in the initiation of noxious cold related DNIC we would have expected to see inhibition of the EMG responses at the 10-min interval. Wolf & Hardy (29) reported decreased pain in subjects that experienced slower cooling of the water bath, therefore it may be that the slow rate of change of skin temperature reflected a stimulus of insufficient magnitude to induce DNIC. Anatomical features identified in the footpads of dogs, including dermal arteriovenous anastomoses, a dermal venous plexus, and a thick layer of subcutaneous adipose tissue (33) have been proposed to be adaptations to a cold environment. It is possible that, despite skin temperatures falling during immersion of the distal limb in ice water, these adaptations prevented tissue temperatures at the depth of cold responsive nociceptors from falling to a degree necessary to represent a moderate or severe nociceptive stimulus.

Acepromazine (34) and alfaxalone (35) exhibit vasodilator activity, which may have countered cold induced vasoconstriction, however administration of vasodilators by Wolf & Hardy (29) did not alter the cold induced pain sensations in their subjects.

Endogenous analgesia following the cold pressor test is reported to persist for at least 3–8 min (18), therefore, had we been able to induce DNIC, we would expect that test stimuli delivered at 5 min intervals would be appropriately timed to demonstrate evidence of suppression of the NWR. We did not deliver test stimuli more frequently, due to concerns that more frequent stimulations would cause sensitization of the NWR. However, work to evaluate the stability of responses to stimuli delivered at 60 s intervals (19), suggest that this frequency does not induce sensitization over a period of 10 min, and therefore more frequent test stimuli could be employed in future DNIC studies, which would increase the sensitivity of the technique to short-lived effects.

We elected to test a paradigm involving sedation of the dogs for both ethical reasons (we were applying electrical stimuli) and also to facilitate the recording of clear EMG responses; additionally, we were interested in developing a paradigm that would be considered acceptable to use in client-owned pet dogs. However, the sedative agents may have affected the evoked NWR. Acepromazine has antagonist activity at dopamine, α_1_ adrenergic, 5-hydroxytryptamine and histamine receptors, in addition to decreasing synaptic uptake of adenosine (36), but is not considered to have analgesic or antinociceptive properties (37). Alfaxalone is reported to be devoid of analgesic activity (38), and its effects are mediated by potentiation of gamma-amino butyric acid (GABA) (39). However, although DNIC is reportedly unaffected by GABA agonists (40, 41) and so acepromazine sedation and alfaxalone sedation or anaesthesia would therefore be expected to provide suitable conditions for investigation of DNIC, GABA receptors do play a role in pain states (42). There are no published studies describing the effects of anaesthetics on cold pressor efficiency and testing characteristics, therefore overall, we cannot exclude the possibility that sedation and anaesthesia have modulated the expected responses. Reassuringly DNIC has been demonstrated in other species utilising a variety of anaesthetic agents to produce unconsciousness. In halothane anaesthetised rats, DNIC has been successfully induced using noxious mechanical force at a number of body sites, noxious heat and electrical stimulation of the tail and intraperitoneal bradykinin (1), whilst in pentobarbitone anaesthetised rabbits cutaneous application of mustard oil to the snout has been used to induce DNIC (2).

In order to safeguard the welfare of animals exposed to a conditioning stimulus, the stimulus should be rapidly terminated, non-tissue damaging and rapidly effective, permitting a short testing time. Given these results, and recent published work, mechanical (15) or ischaemic (14) induced noxious conditioning stimuli may be the most rewarding to investigate further.

## 5 Conclusion

In this study, we found no suppression of electromyographic activity by a cold pressor conditioning stimulus, suggesting DNIC was not activated in this experimental design.

Our recommendations for future investigations of DNIC in dogs include continuing to employ a neurophysiological rather than behavioural measure as the outcome, employing a higher frequency of test stimuli in order to discern short-lived DNIC effects, and investigation of other modalities of conditioning stimuli.

## Data Availability

The raw data supporting the conclusions of this article will be made available by the authors, without undue reservation.
